# Supplementary material for: HIV-1 nuclear import is selective and depends on both capsid elasticity and nuclear pore adaptability
Source: Nat Microbiol. 2025 Jul 7;10(8):1868–85. doi: 10.1038/s41564-025-02054-z (PMC12313523; doi:10.1038/s41564-025-02054-z)
Supplement: Supplementary file 1 — Supplementary Tables 1–13 and captions of Videos 1–10. [file 41564_2025_2054_MOESM1_ESM.pdf]

nature microbiology

Supplementary information https://doi.org/10.1038/s41564-025-02054-z

HIV-1 nuclear import is selective and depends on both capsid elasticity and nuclear pore adaptability

In the format provided by the authors and unedited

Supplementary information

Supplementary table 1. Shape distribution of WT cores in P-CEM cells in multiple states

	Approaching	Docking	Traversing	Imported
Cone-shaped	61	48	14	2
Tube-shaped	9	9	4	8
Total	70	57	18	10

Supplementary table 2. Shape distribution of WT cores in PS-CEM cells in multiple states

	Approaching	Docking	Traversing	Imported
Cone-shaped	220	185	99	26
Tube-shaped	48	32	24	51
Total	268	217	123	77

Supplementary table 3. Shape distribution of E45A cores in PS-CEM cells in multiple states

	Approaching	Docking	Traversing	Imported
Cone-shaped	137	259	7	9
Tube-shaped	17	27	7	11
Total	154	286	14	20

Supplementary table 4. Shape distribution of E45A/R132T cores in PS-CEM cells in multiple states

	Approaching	Docking	Traversing	Imported
Cone-shaped	26	24	22	7
Tube-shaped	6	3	2	12
Total	32	27	24	19

Supplementary table 5. Shape distribution of N74D cores in PS-CEM cells in multiple states

	Approaching	Docking	Traversing	Imported
Cone-shaped	19	14	21	2
Tube-shaped	8	1	5	3
Total	27	15	26	5

Supplementary table 6. Orientation distribution of cone-shaped WT cores in docking and traversing states in PS-CEM cells

	Docking	Traversing
Wide end	93	3
Narrow end	87	89

Supplementary table 7. Orientation distribution of cone-shaped E45A cores in docking and traversing states in PS-CEM cells

	Docking	Traversing
Wide end	128	0
Narrow end	129	7

Supplementary table 8. Orientation distribution of cone-shaped E45A/R132T cores in docking and traversing states in PS-CEM cells

	Docking	Traversing
Wide end	11	2
Narrow end	9	20

Supplementary table 9. Orientation distribution of cone-shaped N74D cores in docking and traversing states in PS-CEM cells

	Docking	Traversing
Wide end	8	1
Narrow end	6	19

Supplementary table 10. Cryo-FIB lamella preparation

**Method**	Correlative milling	Correlative milling
Microscope	Plasma FIB Arctis	Conventional FIB Aquilos 2
Voltage (keV)	30	30
Ion beam source	Argon	Gallium
Sputtering coating prior to milling (seconds)	12	No
GIS coating time (second)	50	30
Bulk milling current	N/A	N/A
Milling current	0.74-2 nA	0.1-0.5 nA
Polishing current	60 pA	30 pA
Sputtering coating post polishing (seconds)	No	No
Fluorescence microscope	iFLM (100 ×)	METEOR (50 ×)
Number of lamellae	46	313

Supplementary table 11. Cryo-ET data collection

**Sample**	Lamellae of WT cores	Lamellae of E45A cores	Lamellae of E45A/R132T cores	Lamellae of N74D cores	All isolated cores on grids	HIV-1 virions	Lamellae of CEM cells	CPSF6 bound to perforated VLP
Microscope	FEI Titan Krios G3	FEI Titan Krios G3	FEI Titan Krios G3	FEI Titan Krios G3	FEI Titan Krios G2	FEI Titan Krios G2	FEI Titan Krios G2	FEI Titan Krios G2
Voltage (keV)	300	300	300	300	300	300	300	300
Detector	Falcon 4i	Falcon 4i	Falcon 4i	Falcon 4i	Gatan K3	Gatan K3	Gatan K3	Falcon 4i
Energy-filter	Selectris X	Selectris X	Selectris X	Selectris X	Gatan BioQuantum	Gatan BioQuantum	Gatan BioQuantum	Selectris X
Slit width (eV)	10	10	10	10	20	20	20	20
Super-resolution mode	No	No	No	No	Yes	No	Yes	No
Physical pixel size (Å/pixel)	1.903/1.94	1.903/1.94	1.903	1.94	0.831	1.34	2.18	1.34
Defocus range (μm)	-3 to -5, increment 0.3	-3 to -5, increment 0.3	-3 to -5, increment 0.3	-3 to -5, increment 0.3	-3 to -4, increment 0.3	-1.5 to -3, increment 0.3	-3 to -5, increment 0.3	-1.5 to -3, increment 0.3
Acquisition scheme	Dose-Symmetric, -52º/52º, -54º/54º, 2º step, group 2	Dose-Symmetric, -54º/54º, 2º step, group 2	Dose-Symmetric, -54º/54º, 2º step, group 2	Dose-Symmetric, -54º/54º, 2º step, group 2	Single-shot micrograph	Dose-Symmetric, -60º/60º, 3º step, group 3	Dose-Symmetric, -54º/54º, 3º step, group 3	Dose-Symmetric, -60º/60º, 3º step, group 3
Total dose (electrons/Å^2)	137.5/132.5	137.5/132.5	137.5	137.5	22	123	37	120
Number of frames	10	10	10	10	59	10	10	10
Number of lamellae	185	72	32	29	N/A	N/A	10	N/A
Number of tomograms/micrographs	759	322	122	179	10,000	58	47	100

Supplementary table 12. Structural determination of WT HIV-1 CA hexamers

**States of HIV-1 WT cores**	Outside	Traversing	Imported
Particle number	11,915	5,545	7,825
Final resolution by gold-standard FSC cut (Å)	11.0	11.7	15.8

Supplementary table 13. Structural determination of NPCs of PS-CEM cells

**Ring moieties**	CR	IR	LR	NR
Particle number	1,121	1,121	1,121	1,121
Final resolution by gold-standard FSC cut (Å)	21.9	28.8	32.1	36.5

Supplementary videos

Supplementary video. 1 | A video showing the tomogram and segmented volume of the nuclear import of three HIV-1 WT cores.

Supplementary video. 2 | A video showing the tomogram of an imported HIV-1 WT core being transported in the nucleus, followed by the depiction of cleaned template matched CA

hexamers (purple orbs), placement of placement of CA pentamers (gold orbs) based on the coordinates of hexamers, and a mapped-back full model.

Supplementary video. 3 | A video showing the tomogram of an imported HIV-1 WT core starting uncoating in the nucleus, followed by the depiction of cleaned template matched CA hexamers (purple orbs), placement of placement of CA pentamers (gold orbs) based on the coordinates of hexamers, and a mapped-back full model.

Supplementary video. 4 | A video showing the tomogram of an imported HIV-1 WT core halfway in uncoating in the nucleus, followed by the depiction of cleaned template matched CA hexamers (purple orbs) and a mapped-back full model.

Supplementary video. 5 | A video showing the tomogram of an imported uncoating HIV-1 WT core releasing viral genome in the nucleus.

Supplementary video. 6 | A video showing the tomogram in video 5 combined with the segmented volume.

Supplementary video. 7 | A video showing the tomogram and template matched coordinates of a deformed NPC and a HIV-1 WT core, followed by mapped-back models.
Supplementary video. 8 | A video showing the tomogram and segmented volume of a just-imported HIV-1 N74D core.

Supplementary video. 9 | A video showing the tomogram and segmented volume of five HIV-1 E45A cores docking and clashing on the NPC.

Supplementary video. 10 | A video showing the tomogram and segmented volume of two HIV-1 E45A/R132T cores in docking and imported states.
