# Supplementary material for: HIV-1 nuclear import is selective and depends on both capsid elasticity and nuclear pore adaptability
Source: Nat Microbiol. 2025 Jul 7;10(8):1868–85. doi: 10.1038/s41564-025-02054-z (PMC12313523; doi:10.1038/s41564-025-02054-z)
Supplement: Supplementary file 2 — Reporting Summary [file 41564_2025_2054_MOESM2_ESM.pdf]

nature portfolio

Corresponding author(s): Peijun Zhang

Last updated by author(s): May 12, 2025

# Reporting Summary

Nature Portfolio wishes to improve the reproducibility of the work that we publish. This form provides structure for consistency and transparency in reporting. For further information on Nature Portfolio policies, see our Editorial Policies and the Editorial Policy Checklist.

## Statistics

For all statistical analyses, confirm that the following items are present in the figure legend, table legend, main text, or Methods section.

| n/a | Confirmed | |
|---|---|---|
| ☐ | ☒ | The exact sample size (*n*) for each experimental group/condition, given as a discrete number and unit of measurement |
| ☐ | ☒ | A statement on whether measurements were taken from distinct samples or whether the same sample was measured repeatedly |
| ☐ | ☒ | The statistical test(s) used AND whether they are one- or two-sided<br>*Only common tests should be described solely by name; describe more complex techniques in the Methods section.* |
| ☒ | ☐ | A description of all covariates tested |
| ☒ | ☐ | A description of any assumptions or corrections, such as tests of normality and adjustment for multiple comparisons |
| ☐ | ☒ | A full description of the statistical parameters including central tendency (e.g. means) or other basic estimates (e.g. regression coefficient) AND variation (e.g. standard deviation) or associated estimates of uncertainty (e.g. confidence intervals) |
| ☐ | ☒ | For null hypothesis testing, the test statistic (e.g. *F*, *t*, *r*) with confidence intervals, effect sizes, degrees of freedom and *P* value noted<br>*Give P values as exact values whenever suitable.* |
| ☒ | ☐ | For Bayesian analysis, information on the choice of priors and Markov chain Monte Carlo settings |
| ☒ | ☐ | For hierarchical and complex designs, identification of the appropriate level for tests and full reporting of outcomes |
| ☒ | ☐ | Estimates of effect sizes (e.g. Cohen's *d*, Pearson's *r*), indicating how they were calculated |

*Our web collection on statistics for biologists contains articles on many of the points above.*

## Software and code

Policy information about availability of computer code

| | |
|---|---|
| Data collection | LAS X version 3.5.9.26787, WebUI version 1.1, AutoTEM 5 version 5.19, METEOR version 1.0, iFLM system version 1.3, Tomography 5 software, EPU version 3.8, FEI TIA version 0.7.1 |
| Data analysis | Leica Application Suite X, FIJI ImageJ version 2.0.0-rc-59/1.51n, Arivis Vision4D version 4.1.2, MotionCor2 version 1.4.0, IMOD version 4.11.1, Prism 10, emClarity version 1.5.0.2, emClarity version 1.5.3.10, RELION version 4.0, UCSF ChimeraX version 1.9, IsoNet version 0.2, MemBrain-seg version 0.0.8, Amira version 2024.2, ArtiaX version 0.6.0, MagpiEM |

For manuscripts utilizing custom algorithms or software that are central to the research but not yet described in published literature, software must be made available to editors and reviewers. We strongly encourage code deposition in a community repository (e.g. GitHub). See the Nature Portfolio guidelines for submitting code & software for further information.

## Data

Policy information about availability of data

All manuscripts must include a data availability statement. This statement should provide the following information, where applicable:

- Accession codes, unique identifiers, or web links for publicly available datasets
- A description of any restrictions on data availability
- For clinical datasets or third party data, please ensure that the statement adheres to our policy

All data needed to evaluate the conclusions in the paper are present in the paper and/or the supplementary information and source data are provided with this

paper. Cryo-EM density maps are deposited in the public data base EMDB under the accession codes: EMD-52887, EMD-52888, and EMD-52889, EMD-53083, EMD-53084, EMD-53085, and EMD-53086.

## Research involving human participants, their data, or biological material

Policy information about studies with human participants or human data. See also policy information about sex, gender (identity/presentation), and sexual orientation and race, ethnicity and racism.

| | |
|---|---|
| Reporting on sex and gender | n/a |
| Reporting on race, ethnicity, or other socially relevant groupings | n/a |
| Population characteristics | n/a |
| Recruitment | n/a |
| Ethics oversight | n/a |

Note that full information on the approval of the study protocol must also be provided in the manuscript.

# Field-specific reporting

Please select the one below that is the best fit for your research. If you are not sure, read the appropriate sections before making your selection.

☒ Life sciences ☐ Behavioural & social sciences ☐ Ecological, evolutionary & environmental sciences

For a reference copy of the document with all sections, see nature.com/documents/nr-reporting-summary-flat.pdf

# Life sciences study design

All studies must disclose on these points even when the disclosure is negative.

| | |
|---|---|
| Sample size | For confocal fluorescence analysis, no statistical calculation was used to predetermine sample size. Approximately 60–160 nuclei were analyzed per condition. This sample size was sufficient to capture consistent and reproducible patterns of nuclei interactions with HIV-1 cores and was in line with sample sizes commonly used in similar confocal imaging studies. For cryo-ET analysis, size of tomograms used for subsequent studies was determined by the quality as reported in the software IMOD version 4.11.1, tomograms with alignment residual error smaller than 1 nm were selected. For subtomogram averaging, no predetermination was calculated, sample size was determined after particle cleaning via the software MagpiEM and RELION version 4.0. For statistical analyses, no sample size was predetermined as samples were extracted from the tomograms of good quality as described above. The detailed sample sizes were included in the corresponding figure legends and were sufficient to carry out faithful analyses as verified by in the software Prism 10. |
| Data exclusions | For confocal fluorescence analysis, CEM nuclei that are either touching the edges of the z-stacks or only partially located within the z-stacks were excluded from the analysis. For analysis of HIV-1 cores and NPCs, data were excluded when cores and NPCs were not clearly visualised within the tomograms. For NPC symmetry analysis, data were excluded when the diameter of the NPC could not be determined. |
| Replication | For confocal fluorescence and cryo-ET imaging of HIV-1 nuclear import, each sample and condition was repeated in at least three independent biological replicates, yielding consistent results. |
| Randomization | For confocal fluorescence, 50 - 160 nuclei were randomly selected for imaging and analysis for each sample and conditions. For cryo-ET analysis, tomograms were not randomly selected, instead, tomograms were selected based on the quality control as described in the Sample size section. Thus, the particles used for subsequent statistical analyses were not randomly selected, but rather extracted specifically from high-quality tomograms. For subtomogram averaging, the dataset were randomly divided into two half sets, as a standard approach implemented in Relion version 4.0 for the final determination of resolution. Although the selection of tomograms was not random, we do not anticipate any consequential relevance to the experiments as the high-quality tomograms were acquired from independent biological replicates (cells). |
| Blinding | For HIV-1 nuclear import assays, confocal imaging and analysis were not blinded, as blinding was not feasible for this experiment. However, we do not anticipate this to affect the conclusions due to the statistically robust dataset. For correlative cryo-FIB and cryo-ET, the workflow was not blinded as the fluorescence was applied to guide the targeted sample preparation and data collection. This was the inherent design of this study, thus no blinding was conducted. |

# Reporting for specific materials, systems and methods

We require information from authors about some types of materials, experimental systems and methods used in many studies. Here, indicate whether each material, system or method listed is relevant to your study. If you are not sure if a list item applies to your research, read the appropriate section before selecting a response.

## Materials & experimental systems

| n/a | Involved in the study | |
|---|---|---|
| ☐ | ☒ | Antibodies |
| ☐ | ☒ | Eukaryotic cell lines |
| ☒ | ☐ | Palaeontology and archaeology |
| ☒ | ☐ | Animals and other organisms |
| ☒ | ☐ | Clinical data |
| ☒ | ☐ | Dual use research of concern |
| ☒ | ☐ | Plants |

## Methods

| n/a | Involved in the study | |
|---|---|---|
| ☒ | ☐ | ChIP-seq |
| ☒ | ☐ | Flow cytometry |
| ☒ | ☐ | MRI-based neuroimaging |

# Antibodies

| | |
|---|---|
| Antibodies used | anti-p24: monoclonal antibody produced from hybridoma 183-H12-5C obtained from the Chesebro laboratory via the NIH HIV Reagent Program |
| Validation | The specificity of the antibody was validated by immunoblotting against purified recombinant CA protein |

# Eukaryotic cell lines

Policy information about cell lines and Sex and Gender in Research

| | |
|---|---|
| Cell line source(s) | Human embryonic kidney (HEK) 293T Lenti-X cells (Takara/Clontech 632180); CD4+ T lymphocyte CEM cells (NIH HIV reagent program/ARP-117) |
| Authentication | Cell lines have not been authenticated |
| Mycoplasma contamination | All the cells were tested negative for mycoplasma contamination |
| Commonly misidentified lines (See ICLAC register) | n/a |

# Plants

| | |
|---|---|
| Seed stocks | n/a |
| Novel plant genotypes | n/a |
| Authentication | n/a |
